# The Hallucinations of Hearing

**Published:** 1915-01-16

**Authors:** 


### MEDICO-PSYCHOLOGY.

### The Hallucinations of Hearing.

One of the commonest mental derangements met with in general practice is that of auditory hallucination, which, always evidencing serious disorder, frequently indicates the presence of a really dangerous form of insanity. The responsibility thrown on the doctor who has to judge as to the significance to be attached to the hallucination in any particular case is a grave one, for he has to arrive at no less a decision than whether the patient who happens to hear "voices" is fit to continue moving among his fellow-men or whether he is in such a state of mind that homicidal or suicidal impulses may be about to develop. As a matter of fact, it is only the medical man who has had some systematic experience of medico-psychological work who is in a position to estimate with any pretence to accuracy the significance of the voices. It cannot be doubted that, in regard to this, very serious mistakes are sometimes made by physicians acting in perfectly good faith; indeed, it is probable that grave errors of this kind will continue to be made until the medical curriculum contains some more comprehensive schedule of psycho-pathology. Of course, hallucinations of hearing may occur in the delirant stage of an acute fever, or during convalescence when, exhausted by disease, the brain cells are in a state of malnutrition and function abnormally. But, considering that such hallucinations commonly disappear when the brain recovers its tone, they have not any serious indication, and so are not likely to bother the practitioner.

### Hallucinatory Voices.

On the other hand, when hallucinations of hearing arise without any obvious cause for wrong functioning of the brain cells, and develop in intensity during the course of years, there soon enters the possibility of their producing disorder of conduct that is likely to be dangerous to the individual or to those about him. Often in the early stages of such mental disturbance the hallucination takes the form of indefinite noises—termed akoasms by some writers—and such do not commonly bring about a state of mind that is perilous. But when these akoasms develop into definite phonemes—that is, into sounds of spoken words—an element of danger must always be suspected. Very rarely is it that hallucinatory "voices" are such as to cause any comfort to their victims, it being certainly true in regard to auditory hallucinations that the willing or unwilling listener seldom hears any good of himself; thus it is that where the patient is unable to resist the evidence of his senses, and believes in the objective origin of the threats or things he hears, it is not long before his resentment is aroused against an imaginary tormentor. Even where the voices are not minatory at first, experience shows that as the disorder advances there is a tendency for them to become threatening, to the end that the harassed victim sooner or later turns upon himself or someone else with the idea of ending his misery or persecution. There are at the present time thousands of persons going about their daily work and conducting themselves in a perfectly orderly manner who are constantly hearing "voices," but it would be impossible to incarcerate these unfortunate persons in institutions for the insane. In many of these cases the evidence of mental derangement rests entirely on the patient's admission that he is subject to auditory hallucinations, and, of course, where he suspects lunacy proceedings may be taken he hides the fact that the voices still worry him. With such people there is indeed always the dangerous possibility that they may refuse to tell of their mental discomforts, and so remain at large until some disaster has been led up to.

### Position of Medical Attendant.

It is very much to be doubted whether in routine medical practice patients of this kind receive the full attention which is their due, and if not it is largely owing to the fact that in dealing with such a disorder, and feeling that he can do very little to relieve it, the doctor considers it unjustifiable to prolong medical attendance under the circumstances. But the practitioner can only fulfil his very obvious duty to the public in these matters by insisting that patients suffering from auditory hallucination present themselves periodically either to himself or to some other physician; for thus only can those who are in responsible positions have any opportunity of estimating from time to time the invalid's position in regard to his fellows. An auditory hallucination that at one consultation may not have any immediately serious meaning may six months later be leading up to a homicidal attack on some unsuspecting individual.

Whilst he cannot invariably advise certification in cases where patients tell him that they hear voices, the practitioner should make it a fixed rule to increase his vigilance whenever he finds that the voices have assumed a threatening or commanding character, and a further rule to take every possible step to secure legal restraint whenever the patient admits that he thinks he can locate the individual or section of society which is responsible for the persecuting voices. For it should be remembered that the moment the sufferer from auditory hallucinations considers that he is the object of systematic conspiracies from a definite quarter, he becomes a dangerous patient and should be treated accordingly. Although it is a matter of some difficulty to benefit patients suffering in this way, and whilst the general tendency is in a downward direction, it is a matter of routine experience that all physical measures that tend to promote the health of the brain and body tend to lessen the intensity of the hallucination, whilst the same may be said of freedom from worry and anxiety. It is a matter of common observation that sleepless nights increase the patient's distress, and by lowering his resistance, as it were, make it all the more difficult for him to accept the assurance of his doctors that the voices he hears have no substantial origin. Consequently by sedative drugs and common-sense mental hygiene the general practitioner can do a great deal more for invalids of this class than perhaps he is always able to understand.

In these days of psycho-therapy it might be supposed that in this form of mental disorder treatment by suggestion or persuasion would be able to do much good, but as a matter of practical experience it would appear that, whilst here and there one finds a case remarkably benefited by such methods, the conditions are generally unfavourable.

Research has not been able to afford the slightest clue as to the physical basis of hallucinations of hearing, although it may not be unreasonable to suppose that for some reason or other a group of nerve-cells in the auditory centres of the brain —probably in the tempero-sphenoidal lobe—takes upon itself an automatic function, and that the individual's powers of inhibition over this group of disordered cells being lost, they proceed to evolve thought-complexes signifying sounds and voices; repeating their unholy records after the fashion of some demon gramophone. Still in some cases a definite physical disturbance is found as the apparently determining cause of the voices heard; that is to say defect of hearing accompanied by tinnitus aurium may be present in the early stages. Thus it is not unreasonable to suppose that in some hyper-sensitive individuals an indefinite buzzing, or some other sound due to physical ear trouble, may have been magnified into a definite hallucination, which has ultimately developed from akoasm to phoneme—that is, from mere sound to actual "voice." From the psychological point of view hallucinations of hearing are explained by saying that certain thought-complexes have become split off from the main stream of thought, and that the imaginary voices heard are symptoms of mental dissociation. But, indeed, the expression of the disorder in terms of this kind, however helpful to the psychologist, scarcely assists the practitioner.

